# Association Between MTHFR rs17367504 Polymorphism and Major Depressive Disorder in Taiwan: Evidence for Effect Modification by Exercise Habits

**DOI:** 10.3389/fpsyt.2022.821448

**Published:** 2022-06-21

**Authors:** Ming-Hong Hsieh, Oswald Ndi Nfor, Chien-Chang Ho, Shu-Yi Hsu, Chun-Te Lee, Cheng-Feng Jan, Pao-Chun Hsieh, Yung-Po Liaw

**Affiliations:** ^1^School of Medicine, Chung Shan Medical University, Taichung City, Taiwan; ^2^Department of Psychiatry, Chung Shan Medical University Hospital, Taichung City, Taiwan; ^3^Department of Public Health and Institute of Public Health, Chung Shan Medical University, Taichung City, Taiwan; ^4^Department of Physical Education, Fu Jen Catholic University, New Taipei, Taiwan; ^5^Research and Development Center for Physical Education, Health, and Information Technology, Fu Jen Catholic University, New Taipei, Taiwan; ^6^Office of Physical Education, Chung Yuan Christian University, Taoyuan City, Taiwan; ^7^Department of Obstetrics and Gynecology, Chung-Kang Branch, Cheng Ching General Hospital, Taichung City, Taiwan; ^8^Medical Imaging and Big Data Center, Chung Shan Medical University Hospital, Taichung City, Taiwan

**Keywords:** epidemiology, anxiety/anxiety disorders, depression, biological markers, genetics

## Abstract

**Background/Aim:**

Recent studies reported that folate supplementation has beneficial effects on major depression. The Methylenetetrahydrofolate reductase (MTHFR) enzyme is crucial in folate metabolism. This population-based study examined the association between MTHFR rs17367504 polymorphism and major depressive disorder based on exercise habits.

**Methods:**

Taiwan Biobank (TWB) provided demographic and genotype data between 2008 and 2015. The biobank participants were Taiwanese aged 30 to 70. Data on major depressive disorder (MDD) were obtained from the National Health Insurance Research Database (NHIRD).

**Results:**

A total of 636 individuals were identified with MDD, whereas 17,298 individuals were considered controls. The associations of MTHFR rs17367504 and exercise with MDD risk were estimated using logistic regression models. The distribution of MTHFR rs17367504 genotype frequencies differed significantly between the MDD and control groups. We found that, compared with the AA genotype, the GG genotype was associated with a significantly increased risk of MDD [adjusted odds ratio (aOR), 1.76; 95% confidence interval (CI), 1.05–2.94; *p* = 0.033]. We found an interaction (*p* = 0.04) between rs17367504 and exercise, a well-known protective factor for MDD. A substantial increase in the risk of MDD was found among those with GG genotypes who did not exercise (aOR, 2.93; 95% CI, 1.66–5.17; *p* < 0.001).

**Conclusions:**

Our findings indicate that MDD is related to MTHFR rs17367504 and exercise, though the mechanisms remain to be determined.

## Introduction

Major depressive disorder is a mental disorder that affects psychosocial functioning and negatively impacts the quality of life. The world health organization (WHO) ranked MDD as the third leading cause of disease burden in 2008 and predicted it would rank first by 2030 (1). MDD treatment and prevention are essential given the high prevalence and societal burden associated with it. The etiology of MDD is unclear; both genetic and environmental factors are believed to play a role (2).

In MDD, elevated homocysteine levels and folate deficiency are consistently observed (3–5). MTHFR is a key enzyme in folate-mediated one-carbon metabolism (6). In folate metabolism, the MTHFR enzyme is responsible for metabolizing folate (5,10-methylenetetrahydrofolate) to 5-methyltetrahydrofolate (5-methyl-THF). The 5-methyl-THF then donates a methyl group for the synthesis of methionine from homocysteine. MTHFR gene polymorphisms result in decreased enzyme activity, resulting in elevated homocysteine and reduced neurotransmitter levels (7). Several single nucleotide polymorphisms (SNPs) in this gene have been reported. Some of the widely investigated MTHFR SNPs, rs1801133 (C677T) and rs1801131 (A1298C) have been associated with reduced enzyme activity (8–10) and increased homocysteine concentrations in blood (11). There is no consistent evidence so far linking C677T and A1298C variants to MDD (12–18). There may be ethnic differences behind these inconsistencies. On the other hand, an epidemiological study has shown that MDD is associated with cardio-metabolic diseases (19). This relationship appears to be influenced by common genetic risk factors (20, 21). A blood-pressure-related SNP such as the MTHFR rs17367504 (22, 23) may also be connected to depression.

Modifiable risk factors have been the focus of MDD prevention. Physical activity, defined as any bodily movement produced by skeletal muscles resulting in energy expenditure (24) is one of the few established modifiable targets for depression prevention. Several meta-analyses of randomized clinical trials (25) and prospective cohort studies (26, 27) have suggested a relationship between physical activity and depression. Exercise can promote adult neurogenesis by increasing levels of β-endorphins, vascular endothelial growth factor, brain-derived neurotrophic factor, and serotonin (28). Therefore, it could be considered to have antidepressant properties.

There is a significant connection between MDD and hypertension (29). Hypertension is highly linked to polymorphism rs17367504 as discussed above. One of the most important factors in the prevention of MDD and hypertension is regular exercise. To our knowledge, exercise-based genetic studies on MDD are rare. Therefore, the present study examined whether exercise can influence the association between MDD and the high blood pressure-associated MTHFR rs17367504 polymorphism among adults in TWB.

## Materials and Methods

### Data Source and Study Participants

This study was conducted using data from TWB participants recruited between 2008 and 2015. Detailed information on the TWB dataset has been published elsewhere (30). In brief, participants were Taiwanese residents aged 30 to 70 years with no history of cancer. They had provided written informed consents before being assessed at biobank centers across Taiwan. The NHIRD contained MDD diagnoses (1998–2015) recorded with the International Classification of Diseases, 9th Revision, Clinical Modification (ICD-9-CM) codes. Genetic data of study participants were linked to their health records in the NHIRD at the Health and Welfare Data Science Center (HWDC) via personal identification numbers. The Institutional Review Board of Chung Shan Medical University approved this study (CS2-16114).

Our study data were gathered from 17,985 participants in TWB who were recruited between 2008 and 2015. Nevertheless, we excluded those with incomplete data (*n* = 36) and missing genotype information (*n* = 15). Following exclusions, there were 17,934 eligible participants. After linking genetic and lifestyle data of these individuals to their health-related records in the NHIRD, we found based on the ICD-9-CM codes that 636 of them had MDD (henceforth referred to as cases) and 17,298 did not (henceforth referred to as controls).

### Data Collection and Genotyping

At enrollment, TWB participants had provided extensively detailed information about their demographic (age, sex, education, body mass index (BMI) and lifestyle habits (physical activity, alcohol drinking, cigarette smoking) during a face-to-face interview and physical examination. Additionally, they provided blood samples for DNA extraction. Genotyping was performed at Academia Sinica using the Axiom™ Genome-Wide TWB 1.0 Array plate (Affymetrix, Santa Clara, CA, USA). So far, a total of 646,783 autosomal SNPs have been genotyped.

### Definition of Study Variables

MDD was the main outcome of this study. A diagnosis of MDD was based on the ICD-9-CM codes 296.2 and 296.3, including at least two outpatient visits or one hospitalization (between 1998 and 2015). Exposure variables included polymorphism rs17367504 and exercise. Participants who had at least three sessions of exercise a week lasting at least 30 min each in the last 3 months were classified as regular exercisers. Using questionnaires, participants in TWB specified exercise patterns that included weight training, swimming, gymnastics, rope jumping, aerobic dance, jogging, hiking, biking, table tennis, Chinese martial arts, “Qigong,” “Taijiquan,” hula hoop, basketball, golf, soccer, strolling, tennis, badminton, and other ball games. Individuals who had not exercised during the 3 months before enrollment were considered nonexercisers.

### Polymorphic Variant and Quality Control

Based on literature reviews, rs17367504 of MTHFR was chosen for analysis. This polymorphism was chosen because of its links with hypertension, a risk factor for MDD. For the quality control, the missing rate was 0.000834, the p-value for the Hardy-Weinberg equilibrium (HWE) test result was 0.1549, and the minor allele frequency (MAF) was 0.1261.

### Statistical Analysis

Categorical variables of the cases and controls were compared using the chi-square test. The MDD risk associated with MTHFR rs17367504 genotypes, sociodemographic characteristics, and lifestyle habits were estimated using logistic regression and reported as ORs and 95% CIs. Adjusted variables included sex, age, education, cigarette smoking, alcohol consumption, exercise, and BMI. To evaluate effect modification by exercise habits, we performed stratified analyses based on the rs17367504 genotypes. We compared the association across different exercise categories (no, yes); and assessed the interaction between the polymorphism and exercise by including the cross-product and the main effect terms in logistic regression models. We performed stratified analyses according to the rs17367504 genotypes. PLINK 1.09 beta and the SAS 9.4 software (SAS Institute, Cary, NC, USA) were used for statistical analyses.

## Results

The frequency distributions for selected characteristics of the 636 MDD patients and 17,298 controls are shown in Table 1. In both the case and control groups, there were a greater proportion of women (63.52 vs. 36.48% and 51.07 vs. 48.93%, respectively). Patients with MDD were older than their controls. The distributions of rs17367504 genotypes in case and control groups were significantly different (*p* = 0.02). The relationship between MDD and the MTHFR rs17367504 variant is shown in Table 2. The risk of MDD was 1.76 times greater for participants with the GG genotype than for those with the AA genotype (aOR, 1.76; 95% CI, 1.05–2.94, *p* = 0.033). Compared to the 30–39 year age group, the aORs for MDD were 1.28, 1.47, and 1.52 in the 40–49, 50–59, and 60–69 age groups, respectively. The adjusted ORs for MDD were 1.61 for smoking, 1.50 for drinking, and 1.52 for BMI<18.5. However, the association between exercise and MDD was not significant (aOR, 0.92; 95% CI, 0.78–1.10).

**Table 1:** Characteristics of patients and controls.

	**Controls**		**MDD patients**		
**Parameters**	**(*****n*** **=** **17,298)**		**(*****n*** **=** **636)**		***p-*value**
	***n***	**(%)**	***n***	**(%)**	
Sex					<0.001
Female	8,834	(51.07)	404	(63.52)	
Male	8,464	(48.93)	232	(36.48)	
Age (years)					0.017
30–39	4,398	(25.42)	128	(20.13)	
40–49	4,643	(26.84)	173	(27.20)	
50–59	4,648	(26.87)	193	(30.35)	
60–70	3,609	(20.86)	142	(22.33)	
BMI (kg/m^2^)					0.008
BMI <18.5	502	(2.90)	30	(4.72)	
18.5 ≤ BMI <24	8,291	(47.93)	323	(50.79)	
24 ≤ BMI <27	5,017	(29.00)	157	(24.69)	
BMI≥ 27	3,488	(20.16)	126	(19.81)	
Education					0.001
Elementary School & below	1,002	(5.79)	34	(5.35)	
Junior/Senior High School	6,700	(38.73)	293	(46.07)	
University & above	9,596	(55.47)	309	(48.58)	
Cigarette smoking					0.142
No	13,062	(75.51)	464	(72.96)	
Yes	4,236	(24.49)	172	(27.04)	
Alcohol drinking					0.020
No	15,510	(89.66)	552	(86.79)	
Yes	1,788	(10.34)	84	(13.21)	
Exercise					0.748
No	10,143	(58.64)	377	(59.28)	
Yes	7,155	(41.36)	259	(40.72)	
*MTHFR* rs17367504					0.020
AA	13,178	(76.18)	497	(78.14)	
AG	3,873	(22.39)	123	(19.34)	
GG	247	(1.43)	16	(2.52)	

*BMI, Body mass index; OR, odds ratio; CI, confidence interval*.

**Table 2:** Association between MDD and study variables.

**Variables**	**aOR (95% CI)**	***p-*value**
*MTHFR* rs17367504		
AA	1.00	
AG	0.84 (0.69–1.03)	0.085
GG	1.76 (1.05–2.94)	0.033
Sex		
Female	1.00	
Male	0.46 (0.37–0.56)	<0.001
Age (years)		
30–39	1.00	
40–49	1.28 (1.01–1.62)	0.042
50–59	1.47 (1.15–1.87)	0.002
60–70	1.52 (1.16–1.99)	0.002
Education		
Elementary School & below	1.00	
Junior/Senior High School	1.43 (0.98–2.08)	0.061
University & above	1.28 (0.88–1.88)	0.199
Cigarette smoking		
No	1.00	
Yes	1.61 (1.28–2.02)	<0.001
Alcohol consumption		
No	1.00	
Yes	1.50 (1.15–1.95)	0.003
Exercise		
No	1.00	
Yes	0.92 (0.78–1.10)	0.355
BMI (kg/m^2^)		
18.5 ≤ BMI <24	1.00	
BMI <18.5	1.52 (1.03–2.24)	0.035
24 ≤ BMI <27	0.86 (0.70–1.05)	0.130
BMI≥27	1.00 (0.81–1.24)	0.998

*aOR, adjusted odds ratio; BMI, Body mass index; OR, odds ratio; CI, confidence interval*.

The relationships between MDD and rs17367504 genotypes were further examined based on exercise habits (Table 3). Compared to AA homozygotes, the GG genotype was significantly associated with an increased risk of MDD among physically inactive individuals (aOR, 2.93; 95% CI, 1.66–5.17). There was no significant association among those who were physically active. There was, however, a significant interaction between rs17367504 and exercise (*p* = 0.042). Furthermore, we conducted subgroup analyses stratified by the genotypes of rs17367504 (Table 4). Regardless of the genotype, the risk estimates for MDD among individuals who exercised were lower than those who did not exercise. GG individuals who exercised had an adjusted OR of 0.11 (95% CI, 0.02–0.55) compared to those who did not. The ORs among those with the AA or AG genotypes were not significant. According to the additive model, the OR for MDD in subjects with the G allele compared to those with the A allele was 0.67 (95% CI 0.46–0.97) in the exercise group and 1.27 (0.93–1.73 in the no-exercise group (Table 5).

**Table 3:** Association of MDD with *MTHFR* rs17367504 genotypes and sociodemographic and lifestyle factors stratified by exercise habits.

**Variable**	**No exercise**		**Exercise**	
	***n*** **=** **10,520**		***n*** **=** **7,414**	
	**aOR (95%CI)**	***p-*value**	**aOR (95%CI)**	***p-*value**
rs17367504				
AA	1.00		1.00	
AG	0.89 (0.68–1.15)	0.357	0.77 (0.56–1.07)	0.118
GG	2.93 (1.66–5.17)	<0.001	0.46 (0.11–1.88)	0.281
Sex				
Female	1.00		1.00	
Male	0.36 (0.27–0.47)	<0.001	0.63 (0.46–0.87)	0.005
Age (years)				
30–39	1.00		1.00	
40–49	1.33 (1.01–1.75)	0.046	1.14 (0.72–1.82)	0.570
50–59	1.55 (1.15–2.10)	0.004	1.22 (0.79–1.87)	0.379
60–70	1.97 (1.39–2.80)	<0.001	1.09 (0.70–1.70)	0.711
Education				
Elementary	1.00		1.00	
School & below				
Junior/Senior	1.50 (0.90–2.51)	0.123	1.33 (0.77–2.29)	0.305
High School				
University	1.36 (0.80–2.31)	0.255	1.20 (0.69–2.09)	0.517
& above				
Cigarette smoking				
No	1.00		1.00	
Yes	2.06 (1.54–2.75)	<0.001	1.10 (0.77–1.58)	0.595
Alcohol consumption				
No	1.00		1.00	
Yes	1.49 (1.05–2.10)	0.024	1.51 (1.01–2.27)	0.044
BMI (kg/m^2^)				
18.5 ≤ BMI <24	1.00		1.00	
BMI <18.5	1.57 (0.99–2.48)	0.056	1.43 (0.69–2.99)	0.338
24 ≤ BMI <27	0.81 (0.62–1.06)	0.119	0.92 (0.69–1.24)	0.593
BMI≥27	1.06 (0.81–1.39)	0.676	0.87 (0.60–1.26)	0.459

*aOR, adjusted odds ratio; BMI, Body mass index; OR, odds ratio; CI, confidence interval*.

**Table 4:** Association of MDD with exercise stratified by *MTHFR* rs17367504 genotypes.

	**rs17367504 (AA)**		**rs17367504 (AG)**		**rs17367504 (GG)**	
**Variable**	***n*** **=** **13,675**		***n*** **=** **3,996**		***n*** **=** **263**	
	**aOR (95%CI)**	***p-*value**	**aOR (95%CI)**	***p-*value**	**aOR (95%CI)**	***p-*value**
Exercise						
No	1.00		1.00		1.00	
Yes	0.96 (0.79–1.16)	0.675	0.98 (0.66–1.46)	0.912	0.11 (0.02−0.55)	0.008
Sex					
Female	1.00		1.00		1.00	
Male	0.44 (0.34–0.55)	<0.001	0.47 (0.30–0.75)	0.002	1.1 (0.32–3.77)	0.883
Age (years)						
30–39	1.00		1.00		1.00	
40–49	1.35 (1.02–1.78)	0.035	1.08 (0.67–1.76)	0.742	1.49 (0.27–8.10)	0.644
50–59	1.71 (1.29–2.25)	<0.001	0.76 (0.43–1.33)	0.336	1.62 (0.31–8.55)	0.571
60–70	1.63 (1.20–2.22)	0.002	1.03 (0.56–1.87)	0.932	5.09 (0.98–26.33)	0.053
Education						
Elementary School & below	1.00		1.00		1.00	
Junior/Senior High School	1.64 (1.06–2.55)	0.027	0.88 (0.40–1.96)	0.761	0.93 (0.15–5.88)	0.940
University & above	1.48 (0.95–2.31)	0.087	0.82 (0.36–1.85)	0.628	0.77 (0.11–5.16)	0.784
Cigarette smoking						
No	1.00		1.00		1.00	
Yes	1.62 (1.25–2.10)	<0.001	1.81 (1.11–2.97)	0.018	0.43 (0.08–2.32)	0.328
Alcohol consumption						
No	1.00		1.00		1.00	
Yes	1.58 (1.18–2.12)	0.002	1.19 (0.64–2.22)	0.582	3.55 (0.54–23.57)	0.190
BMI (kg/m^2^)						
18.5 ≤ BMI <24	1.00		1.00		1.00	
BMI <18.5	1.50 (0.96–2.34)	0.073	1.86 (0.82–4.20)	0.137	<0.01 (<0.01–999)	0.978
24 ≤ BMI <27	0.87 (0.69–1.09)	0.214	0.88 (0.55–1.40)	0.597	0.48 (0.11–2.01)	0.313
BMI≥27	0.96 (0.75–1.23)	0.765	1.12 (0.70–1.79)	0.648	0.95 (0.24–3.80)	0.940

*aOR, adjusted odds ratio; BMI, Body mass index; OR, odds ratio; CI, confidence interval*.

**Table 5:** Association of SNPs (rs1801131, rs1801133, and rs17367504) with MDD based on additive, dominant, and the recessive models stratified by exercise habits.

**Model**	**Genotype**	**No Exercise**		**Exercise**	
		**aOR (95%CI)**	***p-*value**	**aOR (95%CI)**	***p-*value**
Additive model					
rs1801131	G vs. T	0.82 (0.63–1.06)	0.133	1.17 (0.88–1.54)	0.282
rs1801133	A vs. G	0.92 (0.77–1.09)	0.337	1.00 (0.82–1.23)	0.980
rs17367504	G vs. A	1.27 (0.93–1.73)	0.135	0.67 (0.46–0.97)	0.032
Dominant model					
rs1801131	GG+GT vs. TT	0.86 (0.65–1.16)	0.330	1.20 (0.87–1.65)	0.275
rs1801133	AA+AG vs. GG	0.88 (0.71–1.09)	0.237	1.02 (0.79–1.32)	0.900
rs17367504	GG+GA vs. AA	1.07 (0.77–1.49)	0.681	0.66 (0.45–0.98)	0.039
Recessive model					
rs1801131	GG vs. GT+TT	0.57 (0.29–1.13)	0.107	1.02 (0.52–1.98)	0.960
rs1801133	AA vs. AG+GG	1.01 (0.67–1.51)	0.968	0.97 (0.61–1.55)	0.901
rs17367504	GG vs. GA+AA	4.63 (1.98–10.83)	<0.001	0.49 (0.11–2.22)	0.352

*The aORs with their 95% CIs were estimated by a multiple logistic regression model after controlling for sex, age, education, cigarette smoking, alcohol drinking, exercise, and BMI*.

## Discussion

As far as we know, this is the first study to evaluate MDD, polymorphism rs17367504 and exercise in the adult population. We found that the MTHFR rs17367504-GG genotype was associated with 1.76 times greater odds of MDD compared to the AA genotype. In contrast to previous studies (26, 27), we found that exercise was not associated with MDD. However, there was evidence of an interaction between rs17367504 and exercise. Following stratification, we found that the GG genotype remained associated with MDD risk, particularly among adults who did not do any physical exercise.

MTHFR is an essential enzyme in the folate-mediated one-carbon metabolic pathway (6). The G allele of MTHFR rs17367504 has also been associated with elevated blood pressure (22, 23, 31) or hypertension, which causes stroke. A growing body of evidence suggests that stroke and MDD can be caused by the same genetic variation (21). We report a novel link between the blood pressure-associated locus (rs17367504) and MDD, and this adds to the evidence suggesting that the MTHFR gene plays a role in the development of the disease. This suggests that both MDD and hypertension may be influenced by this gene. Studies have identified possible biological mechanisms underlying depression and hypertension/stroke, including altered inflammatory responses, dysregulation of lipid and lipoprotein metabolism, and imbalanced neurotransmitters (32, 33). These mechanisms, however, remain poorly understood. Research has suggested that low MTHFR activity or folate deficiency may be associated with depression and other psychiatric disorders (34, 35). According to Wan et al., reduced MTHFR activity increases homocysteine and decreases DNA methylation-dependent methyl donors leading to hypomethylation. Despite this, the effectiveness of MTHFR polymorphisms in explaining specific psychiatric symptoms, including major depression, remains uncertain (35). Therefore, functional studies are necessary to explain the effect of genetic polymorphism on MDD.

Research studies have focused largely on the MTHFR missense SNP, rs1801133 (C677T) and regulatory SNP, rs1801131 (A1298C) (12–18), and have found associations between the minor alleles and MDD. Notably, the 677C/T polymorphism is associated not only with hypertension (36), but also with cardiovascular disease (37). Neither rs1801133 nor rs1801131 is in linkage disequilibrium with MTHFR rs17367504. Until now, no studies have determined what role the MTHFR rs17367504 polymorphism plays in MDD pathogenesis.

It is well known that physical activity may protect against MDD (38). Due to an interaction between MTHFR rs17367504 and exercise, we performed stratified analyses and found that the GG genotype of MTHFR rs17367504 combined with no exercise was associated with a 2.93-fold increased risk of MDD (Table 3). Besides, we also performed group analyses according to rs17367504 genotypes. The overall data indicated that participants who exercised had a decreased risk of MDD (Table 4). However, neither AA nor AG genotypes showed substantial association with MDD, suggesting that they might not be factors for the disease. We found that exercise was more protective in those with the GG genotype. Some estimates failed to reach significance, probably because of the small sample size of the subgroups. We do not understand the biological mechanisms behind the the MTHFR rs17367504 and exercise interaction. Exercise may have positive physiological effects on measures of adult neurogenesis such as increased β-endorphins, vascular endothelial growth factor, brain-derived neurotrophic factor, and serotonin, all of which may improve mood (28). There is also a consensus that increased plasma homocysteine is a risk factor for MDD (39). Human and animal studies demonstrated that exercise alters homocysteine levels in blood (40–45). We propose that the MTHFR rs17367504-GG genotype may moderate MTHFR activity, leading to a substantial increase in plasma homocysteine. Exercise decreases homocysteine levels in the blood. The association between MTHFR rs17367504 and MDD might have been modified by individual exercise habits, which have been linked to altered homocysteine levels as stated above. Additionally, functional MTHFR SNPs, rs1801133 and rs1801131 were included in our genetic models as they are associated with reduced enzyme activity and homocysteine levels as previously noted. However, regardless of exercise status or the model used, neither appeared to be associated with MDD (Table 5).

The larger sample size of this population-based study is its major strength. Next, we linked our available data sources to provide context for the observed associations. However, we acknowledge some limitations: First, we used a cross-sectional study design and the observed relationships cannot be confirmed as causal. Second, exercise intensity and frequency are emerging as the most important factors in modifying disease. Nevertheless, we did not have measures of intensity or energy expenditure in our database. Third, folic acid consumption is associated with MTHFR protein and activity levels. However, homocysteine and folate levels were not included in this study as such information was not available in the biochemistry data in TWB. Next, antidepressant medications were not specifically investigated in the TWB. Our study, therefore, could not include these medications. Furthermore, the system at the ministry of health and welfare does not support the software for haplotype analysis, so we were unable to present data according to haplotypes. Lastly, TWB consists of samples from individuals aged 30 to 70 and is representative of the general population. Therefore, our findings may not apply to individuals younger than 30 or older than 70.

## Conclusion

To sum up, our data indicate that sedentary lifestyle may increase MDD risk among MTHFR rs17367504-GG individuals in Taiwan. These findings might be useful in developing public health strategies aimed at reducing or preventing MDD.

## Data Availability Statement

The original contributions presented in the study are included in the article/supplementary material, further inquiries can be directed to the corresponding author.

## Ethics Statement

This study was approved by the Chung Shan Medical University Institutional Review Board (CS1-20009). All methods were carried out in accordance with relevant guidelines and regulations. Also, informed consent was obtained from all the participants in the study.

## Author Contributions

M-HH, C-TL, ON, S-YH, C-CH, C-FJ, P-CH, and Y-PL conceptualized and designed the study. S-YH, C-CH, ON, C-TL, C-FJ, P-CH, and Y-PL managed, analyzed, and interpreted data. M-HH and ON drafted the manuscript. P-CH, C-TL, S-YH, C-CH, C-FJ, and Y-PL critically reviewed the manuscript. All authors read and approved the submitted version.

## Funding

This work was supported by the Ministry of Science and Technology (MOST-110-2121-M-040-002).

## Conflict of Interest

The authors declare that the research was conducted in the absence of any commercial or financial relationships that could be construed as a potential conflict of interest.

## Publisher's Note

All claims expressed in this article are solely those of the authors and do not necessarily represent those of their affiliated organizations, or those of the publisher, the editors and the reviewers. Any product that may be evaluated in this article, or claim that may be made by its manufacturer, is not guaranteed or endorsed by the publisher.
